# Opinion: Opportunities and Limitations of Machine Vision for Yield Mapping

**DOI:** 10.3389/frobt.2021.627280

**Published:** 2021-02-25

**Authors:** John K. Schueller

**Affiliations:** University of Florida, Gainesville, FL, United States

**Keywords:** yield mapping, machine vision, artificial intelligence, limitations, sensing

## Introduction

Precision agriculture has been one of the most exciting innovations in agriculture during the recent decades. Precision agriculture responds to the inherent spatial variability in crop production, often by machine automation. There were significant and diverse efforts already about thirty years ago (Schueller, 1992). The economic and environmental advantages are widely known. Research, technology transfer, commercialization, and adoption has led to widespread precision agriculture usage, but not complete adoption. Although the limitations to precision agriculture in general have long been identified (e.g., Schueller, 1996) they have only partially been surmounted.

Many view yield the generation of yield maps as the most important precision agriculture technology. It provides the basic information on agricultural production which can subsequently be used for strategic and tactical decisions, such as the variable rate applications of inputs including irrigation, fertilizer, and pesticides. The adoption of yield mapping has been gradual, but significant adoption has been seen and documented (e.g., Schimmelpfenning, 2019).

Of course, the determination of yields as a function of geographic location has long been practiced in both the private and public sectors (e.g., Central Intelligence Agency, 1954). As technologies were developed, satellites became widely used for this purpose (e.g., Erickson, 1984). While widespread and useful for political, economic, and military purposes, such maps are large scale and usually not considered yield mapping from an on-farm within-field perspective.

Since the 1980s (e.g., Searcy et al., 1989) yield mapping has been demonstrated on farms by instrumenting harvesting machines, particularly grain combines, to measure the mass or volume of the harvested crop just after it has been harvested. This remains the dominant yield mapping method. For hand-harvested and some machine-harvested crops, yield maps are also sometimes made after harvesting (e.g., Schueller et al., 1999) before transportation and storage.

The biggest problem of such yield mapping during or after harvest is that the yield map information is not available before harvesting. In addition, the harvesting process often aggregates harvested crop over spatial areas or times, depending upon the spatial and/or temporal dynamics of the harvesting processes and machines. This limits the accurate resolution of the resulting yield maps. Some yield measurement techniques (e.g., impact crop flow sensors) may also have negative effects on the harvested crop. Or the techniques may need to be modified for changes in crops or conditions.

## Machine Vision for Yield Mapping

Machine vision has the potential to be very useful in on-farm in-field yield mapping (e.g., Häni et al., 2020). It can give sufficient yield resolution in a timely manner to enable better tactical and strategic decisions. There are also significant advantages in machine vision being noncontact.

Machine vision techniques have also been used to produce maps of estimated yield before the harvesting operations (e.g., Gan et al., 2018). This can be very useful to perform late-season corrective actions in the crop production. It is also useful to scheduling harvesting, including arranging labor and machinery availability. In addition, such pre-harvest yield maps can guide crop marketing decisions.

Machine vision technologies also generally can be more detailed than other yield mapping technologies. They can make yield estimates on individual plants or small areas of plants. The yield estimates can therefore be done with less or no spatial or temporal aggregation.

Machine vision technologies, whether preharvest or at-harvest, can have the additional advantage of simultaneously evaluating the characteristics or quality of the agricultural product. For example, the shape, size, or color can be determined for grading purposes. Another example is that defects and pest infestations can be identified.

The traditional sensing methods are usually embedded on instrumented harvesting or transportation machines. Repair or modification can be disruptive or costly in some situations. Machine vision techniques typically utilize cameras attached to the machine. They usually can be easily replaced or repaired. And the cameras’ performance and techniques can often be changed with just software program changes.

Machine vision equipment techniques can be applied to a wide range of field machines utilized in crop production, whether ground-based or aerial. Particular cameras can be fit to large and small machines and often irrespective of the machine manufacturer and model. The equipment and techniques are applicable to a wide variety of crops and commodities.

## Limitations of Machine Vision Yield Mapping

Yield mapping by machine vision has some significant difficulties, as do all sensing techniques. The interactive combination of physical, chemical, and biological variations in agricultural crops makes yield mapping challenging. These challenges are compounded by the variations in environmental and weather variables, such as precipitation, temperature, and humidity. Weeds, insects, and diseases can also complicate matters and cause further performance degradations. While these factors can affect multiple yield mapping sensing techniques, they can be especially impactful when using machine vision techniques as the harvesting process often removes or reduces the complications for other technologies.

There are some factors which can especially affect machine vision yield mapping performance. Perspective and line-of-sight may be particularly problematic for machine vision attempts to measure yield. Obscured objects or portions of objects are often a problem in machine vision applications before harvest. This is particularly relevant in machine vision for yield mapping due to line-of-sight limitations where measured yield items, other crop organs, and weeds can obscure further crop that needs to be measured to get an accurate yield.

There have been a substantial number of attempts to use machine vision to do yield mapping. These attempts have been useful in that they demonstrate that machine vision has a great potential for noncontact yield mapping. They have also demonstrated many of the problems, issues, parameters, and characteristics of the crops and their environments as seen in their effects upon the performance of machine vision yield mapping systems.

Despite all the knowledge and experience that has been gained, there is a need for further understanding to maximize the potential for machine vision and to move it to successful commercialization. One problem with the general trend of many previous studies is that the research and developments have been conducted for just a specific set of circumstances. Given all the complications (variations in lighting, pests, crops, equipment, etc.), the studies tend to be empirical. So the knowledge is not very applicable to other sets of circumstances. I believe there is a need for trying to develop more rational understandings and models.

There needs to be more fundamental understandings. For example, if particular wavelengths identify the crop product (such as fruit) over other items (such as crop stems or weeds), there should be an understanding of why that is so from a physical, chemical, or biological viewpoint. There needs to be accurate and easily-used models to allow the accurate calculation of yields in diverse crops and conditions based upon the images that are captured by commercial sensors.

One of the potential ways to do this would be developing an understanding of interactions between variables and ways to counteract covariants. For example, in earlier work (MacArthur et al., 2006) we mapped citrus yield by identifying visible orange fruit with overhead drone cameras. A reasonable approximation of the total visible and invisible fruit in healthy trees could be made by multiplying the number of visible fruit by a correction factor. However, if that some correction factor was used on unhealthy trees with less foliage, the number of fruit would be overestimated. So a more robust algorithm must assess the tree size and health. Models are needed to provide the correction factor based upon tree species, health, size, and vigor. And these models may change during the growing season as if machine vision techniques are to be used during the growing season.

Artificial intelligence techniques, such as machine learning and deep learning, may have a capability to handle the complex situations that machine vision yield mapping systems encounter. But again, the difficulty is extending and generalizing, especially if the inference space of the training is limited. Either the inferences spaces, and the subsequent artificial intelligence training, must be increased or rational models developed.

## Conclusion

Machine vision has a great potential for yield mapping, particularly given the great contempoary advances in the machine vision sensors, computational processing speeds, storage economy, and artificial intelligence techniques that can now be utilized. However, much more work is needed to get reliable, accurate, and widespread satisfactory machine vision yield mapping performance. Going beyond the empirical to get rational understandings would be useful. There must be understandings of the reasons for machine vision yield mapping performances and problems so that designs can be developed and refined for widespread commercial success.

## References

[B1] Central Intelligence Agency (1954). Estimate of the 1953 grain production in the Soviet Bloc. Intelligence Memorandum 395. Washington, D.C., United States: Central Intelligence Agency, United States Government.

[B2] Erickson J. D. (1984). “Chapter 8: The LACIE experiment in satellite aided monitoring of global crop production,” in The role of terrestrial vegetation in the global carbon cycle: measurement by remote sensing. Editor Woodwell G. M. (New York, NY: John Wiley & Sons)

[B3] Gan H. Lee W. S. Alchanatis V. Ehsani R. Schueller J. K. (2018). Immature green citrus fruit detection using color and thermal images. Comput. Electron. Agric. 152, 117–125. 10.1016/j.compag.2018.07.011

[B4] Häni N. Roy P. Isler V. (2020). A comparative study of fruit detection and county methods for yield mapping in apple orchards. Field Robot. 37 (2), 263–282. 10.1002/rob.21902

[B5] MacArthur D. K. Schueller J. K. Lee W. S. Crane C. D. MacArthur E. Z. Parsons L. R. (2006). “Remotely-piloted helicopter citrus yield map estimation,” in ASABE Paper No. 063096ASABE. Annual international meeting; Portland, OR, United States; July 9–12.

[B6] Schimmelpfenning D. (2019). “Chapter 2.11—precision agriculture,” in Agricultural resources and environmental indicators, 2019. Editors Hellerstein D. Vilorio D. Ribaudo M. (Washington, D.C., United States: United States Department of Agriculture). Economic Information Bulletin Number 208.

[B7] Schueller J. K. (1992). A review and integrating analysis of spatially-variable control of crop production. Fertil. Res. 33, 1–34. 10.1007/bf01058007

[B8] Schueller J. K. (1996). Communication to the Editor: impediments to spatially-variable field operations. Comput. Electron. Agric. 14 (2/3), 249–253. 10.1016/0168-1699(95)00051-8

[B9] Schueller J. K. Whitney J. D. Wheaton T. A. Miller W. M. Turner A. E. (1999). Low-cost automatic yield mapping in hand-harvested citrus. Comput. Electron. Agric. 23 (2), 145–153. 10.1016/s0168-1699(99)00028-9

[B10] Searcy S. W. Schueller J. K. Bae Y. H. Borgelt S. C. Stout B. A. (1989). Mapping of spatially-variable yield during grain combining. Trans. ASAE 32 (3), 826–829. 10.13031/2013.31077

